# Strain-free MoS_2_/ZrGe_2_N_4_ van der Waals Heterostructure: Tunable Electronic Properties with Type-II Band Alignment

**DOI:** 10.1021/acsomega.4c03193

**Published:** 2024-07-05

**Authors:** Mustapha Driouech, Amrita Mitra, Caterina Cocchi, Muhammad Sufyan Ramzan

**Affiliations:** † Institut für Physik, Carl von Ossietzky Universität, 26129 Oldenburg, Germany; ‡ Center for Nanoscale Dynamics (CeNaD), Carl von Ossietzky Universität, 26129 Oldenburg, Germany

## Abstract

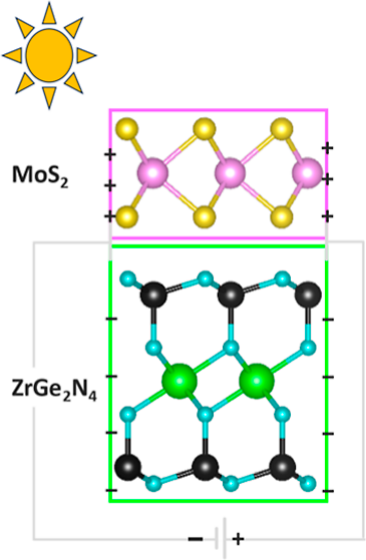

Vertically stacked van der Waals heterostructures (vdW-HS) amplify the scope of 2D materials for emerging technological applications, such as nanodevices and solar cells. Here, we present a first-principles study on the formation energy and electronic properties of the heterobilayer (HBL) MoS_2_/ZrGe_2_N_4_, which forms a strain-free vdW-HS thanks to the identical lattice parameters of its constituents. This system has an indirect band gap with type-II band alignment, with the highest occupied and lowest unoccupied states localized on MoS_2_ and ZrGe_2_N_4_, respectively. Biaxial strain, which generally reduces the band gap regardless of compression or expansion, is applied to tune the electronic properties of the HBL. A small amount of tensile strain (>1%) leads to an indirect-to-direct transition, thereby shifting the band edges at the center of the Brillouin zone and leading to optical absorption in the visible region. These results suggest the potential application of HBL MoS_2_/ZrGe_2_N_4_ in optoelectronic devices.

## 1 Introduction

Layered materials have attracted considerable attention of the scientific community due to their remarkable electronic and optical properties.^1−7^ Various classes of two-dimensional (2D) sheets have been realized experimentally^8−16^ and many more have been theoretically predicted.^17−20^ Among them, semiconducting transition metal dichalcogenides (TMDCs) with chemical formula MX_2_ (where M = Mo and W, and X = S, Se, and Te) have been extensively studied thanks to their unique optoelectronic properties.^13,15,21−25^ Several methods have been proposed to tune the electronic, optical, and structural properties of 2D materials.^26−34^ In particular, stacking different 2D layers on top of each other to form van der Waals heterostructures (vdW-HS) has amplified the catalogue of available materials with diversified properties including type-II band alignment, ultrafast charge transfer, and tunable negative differential resistance.^32,35−37^ On top of this, individual layers with different lattice parameters can give rise to complex patterns and, in some cases, to moiré superlattices.^38−41^

Although vdW-HS carry fascinating physics, treating them from first principles can be computationally prohibitive due to the enormous size of the supercells that are often required to simulate them strain-free. Some strategies have been proposed to reduce these numerical efforts. For example, the so-called “two-step approach” allows one to predict the electronic structure of vdW-HS by performing two separate calculations adopting the lattice parameters of each constituent alone.^32,37^ This method offers reasonable accuracy but is applicable only to systems with a type-II level alignment; also, it neglects the impact of residual strain on the structural and electronic properties of the heterostructure. The increasing number of available 2D materials continuously enhances the amount of vdW-HS that can be explored computationally, making the above-mentioned limitations particularly restraining.

The recent discovery of MoSi_2_N_4_,^42^ a new 2D semiconductor with *P*6̅*m*2 space group and a thickness of seven atomic planes, has stimulated the development of a new class of 2D materials with chemical formula MA_2_Z_4_,^42−48^ where M is a transition metal (from group IVB, VB, and VIB), while A and Z are semimetallic and nonmetallic species of group IVA and VA, respectively. Recently, vdW-HS including monolayer MoSi_2_N_4_ and other 2D materials, such as MoS_2_, graphene, and NbS_2_, have been studied theoretically.^49−53^ However, all of these systems suffer from lattice mismatch and require very large supercells to be simulated strain-free.

Interestingly, a computationally predicted member of the MA_2_Z_4_ family, ZrGe_2_N_4_,^42^ has the same lattice parameter as MoS_2_. ZrGe_2_N_4_ has a direct band gap of 0.85 eV at Γ and is an excellent thermoelectric material due to its low thermal conductivity.^54^ Due to these intriguing characteristics and the lattice matching with MoS_2_,^21^ the heterobilayer (HBL) formed by ZrGe_2_N_4_ and MoS_2_ represents an ideal platform to study from first principles a strain-free vdW-HS. In particular, it allows for a systematic assessment of the effects of strain, distributed equally on both layers, on the electronic properties of this interface. Hence, we chose this material combination to investigate the effects of strain on the HBL without spurious contributions arising from a lattice mismatch. It should be noticed that both constituting materials exhibit the 1T and 2H phase. However, 1T-MoS_2_ is metastable and shows instabilities at room temperature.^55^ For ZrGe_2_N_4_, both phases are predicted to be stable,^56^ but more studies focus on the 1T phase due to its better thermoelectric performance.^54^

Based on this evidence, we focus herein on the strain-free 2H-MoS_2_/1T-ZrGe_2_N_4_ HBL studying its formation energy and electronic structure. After the characterization of the pristine system, which has an indirect band gap and a type-II level alignment, we simulate it under both tensile and compressive biaxial strain with a focus on the interplay between strained lattices and charge redistribution between the two layered materials. We find that values of tensile strain >1% cause an indirect-to-direct band gap transition preserving the type-II level alignment with optical absorption peaks predicted in the visible range, suggesting intriguing perspectives for the MoS_2_/ZrGe_2_N_4_ vdW-HS as a suitable candidate for optoelectronic devices and solar cell applications.

## 2 Computational Methods

The results presented in this work are obtained from density functional theory (DFT)^57^ using the Vienna ab initio simulation package (VASP)^58^ implementing the projector augmented wave method.^59^ The HBL is modeled in an unit cell containing a total of 10 atoms (three from MoS_2_ and seven from ZrGe_2_N_4_) and a vacuum layer of 30 Å in the nonperiodic direction to avoid spurious interactions between periodic images. In the structural optimization step and in the evaluation of the charge-density distribution, the exchange correlational potential is treated at the level of the generalized gradient approximation proposed by Perdew, Burke, and Ernzerhof (PBE)^60^ and supplemented by Grimme’s DFT-D3 correction^61^ to account for dispersive interactions. Spin–orbit coupling (SOC) is included in all calculations except for the postprocessing runs to visualize the wave function distribution in real space: in those cases, we checked that SOC did not induce any perceivable change in the plots. An 18 × 18 × 1 k-point mesh and an energy cutoff of 520 eV are adopted for volume and structural relaxation with convergence thresholds of 1 × 10^–8^ eV for the energy and 10 meV Å^–1^ for the interatomic forces. The electronic structure is subsequently computed with the range-separated hybrid functional by Heyd Scuseria, and Ernzerh (HSE06).^62^ Due to higher computational costs, in these runs, the k-point mesh is halved after checking the convergence of the electronic structure, see Figure S2. Biaxial strain defined as , where *a*(*a*_0_) corresponds to the lattice constant of the strained (unstrained) heterostructure, is applied adopting positive (negative) values for tensile (compressive) strain up to ±4%. Crystal structures and wave-function plots are visualized using VESTA.^63^

## 3 Results and Discussion

### 3.1 Structural Properties

**Figure 1:**
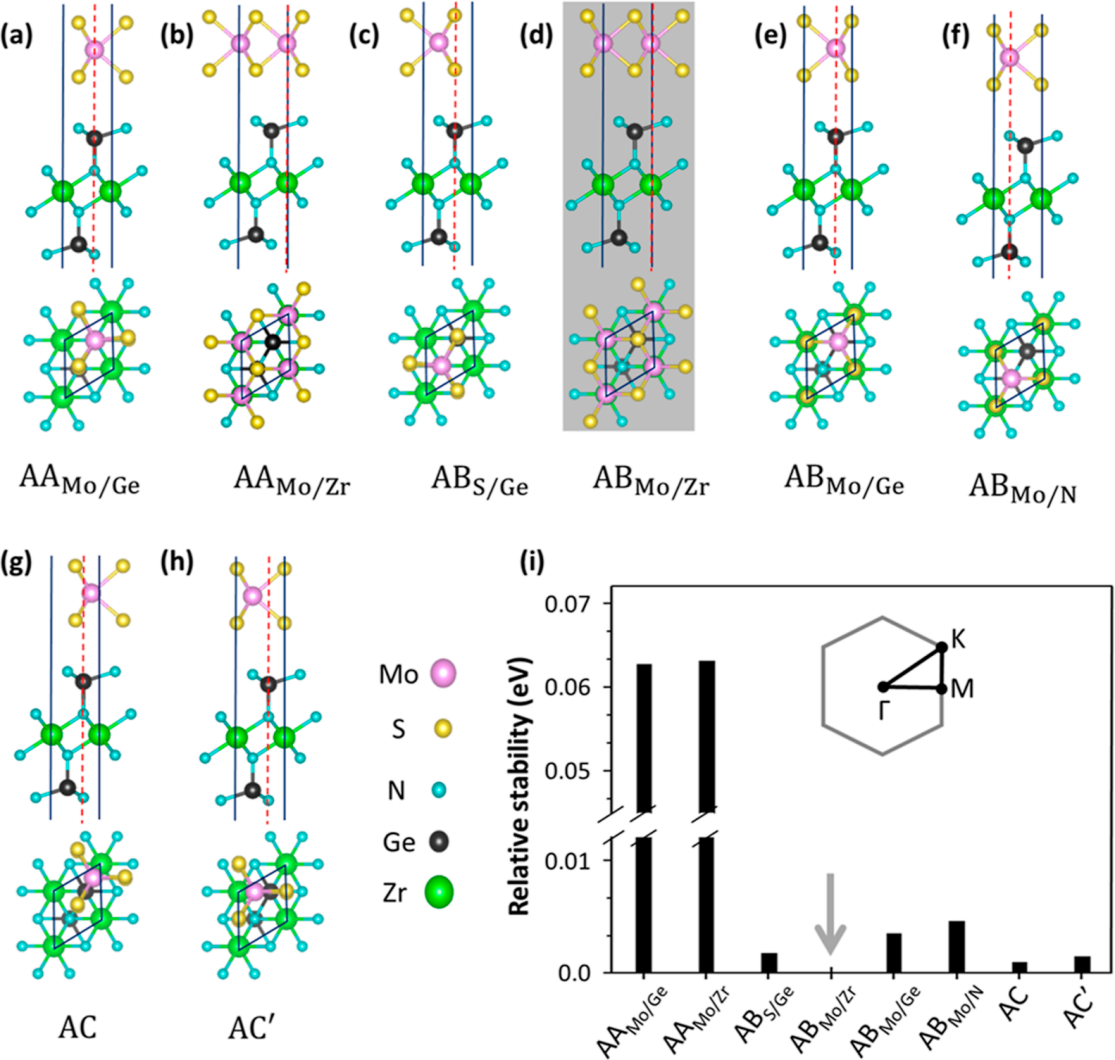
Side and top views of the HBL MoS_2_/ZrGe_2_N_4_ in the (a,b) AA, (c–f) AB, and (g,h) AC stacking configurations, with their point of reference for the stacking indicated by the dashed red lines and their primitive unit cells marked in black. (i) Relative stability of the considered configurations compared to the most stable structure AB_Mo/Ge_ (gray arrow) whose energyis set to zero. The inset shows the Brillouin zone of the investigated HBL with the relevant high-symmetry points and the path connecting them highlighted in bold.

In this study, we consider the vdW-HS formed by monolayer ZrGe_2_N_4_ in the 1T-phase, where the N atoms form a distorted octahedron with Zr atoms in the middle (see Figures 1 and S1), and a single MoS_2_ sheet in the 2H phase, where Mo is surrounded by six S atoms forming a centrosymmetric trigonal prism.^54,64^ Both monolayers are initially optimized and the resulting lattice parameters (*a* = 3.17 Å for both) are in good agreement with earlier works performed at the same level of theory.^65^ Optimized MoS_2_ has bond length *d*_Mo–S_ = 2.41 Å, while in ZrGe_2_N_4_, three different bond lengths are relevant: *d*_Zr–N_ = 2.18 Å, *d*_Ge–N_ (in-plane) = 1.91 Å, and *d*_N–Ge_ (out of plane) = 1.87 Å; they all match with earlier reports.^54,56,66^ We checked the dynamical stability of computationally predicted ZrGe_2_N_4_ by calculating its phonon spectrum (see Figure S7), which does not feature any imaginary frequency. The thermal stability of this compound in the 1T phase was previously demonstrated with molecular dynamics simulations up to 2000 K.^54^

We build the MoS_2_/ZrGe_2_N_4_ HBL considering three stacking arrangements, labeled as AA, AB, and AC, see Figure 1a–h. Due to the different types of atoms included in the vdW-HS, several configurations emerge for each stacking order. AA structures are obtained by placing Mo atoms on top of Ge atoms (labeled as AA_Mo/Ge_) and Zr atoms (AA_Mo/Zr_). In the AB stacking, S atoms are on top of Ge, while N is at the center of the hexagon formed by MoS_2_. In the AC and AC′ stackings, Ge atoms are on top of the Mo–S bonds with their projection closer to Mo. The interlayer distance *d* between the S and N atoms is in the range of 2.97–3.38 Å, see Table S1, and it is shortest in the AB-stacked structures.

We assess the relative stability of the considered vdW-HS in terms of their total energies computed from DFT, see Figure 1i, since all materials have the same number and types of atoms. The AB_Mo/Zr_ HBL (Figure 1d), is the most stable structure: for visualization purposes, its energy is set to zero in Figure 1i and marked by a gray arrow. The HBL with AC stacking (Figure 1g) is energetically very close to the AB_Mo/Zr_ one with an energy difference of 1 meV only. The AC′ (Figure 1h) and AB_S/Ge_ (Figure 1c) HBL exhibit energies that are only 2 meV higher than the minimum. The remaining AB configurations, AB_Mo/Ge_ and AB_Mo/N_, are less stable by about 5 meV, while larger energies (>60 meV) are found for AA_Mo/Ge_ and AA_Mo/Zr_. The small differences in the formation energies of the considered stackings, except for the AA configurations, indicate that this structural parameter does not play a crucial role in the formation of the HBL. This assumption is supported by the fact that all the bond lengths in each constituent are identical regardless of the stacking, see Table S1. In the following, we continue with the analysis of the electronic properties, focusing on the most stable structure AB_Mo/Zr_. We confirmed its dynamical stability by calculating its phonon dispersion (see Figure S7) which does not show any imaginary frequency.

A deeper analysis of the structural properties of the considered vdW-HS shows that for all stackings the Mo–S bond in MoS_2_ (*d*_Mo–S_) is slightly reduced in the HBL compared to the isolated sheet, see Table S1, as a consequence of the vdW interactions occurring at the interface with ZrGe_2_N_4_. Interestingly, this effect is not present in ZrGe_2_N_4_ where the interatomic distances remain unchanged compared with the isolated material. By stretching the HBL in the AB_Mo/Zr_ configuration, the interlayer distance increases to 3.01 Å with 2% strain but decreases to 2.82 Å by enhancing strain to 4%, see Table S2. Conversely, upon compression, the interlayer distance first decreases by 0.04 Å with −1% strain, it is equal to the value in the unstrained system under −2% strain (2.97 Å), and then increases to 3.02 Å with larger strain. A similar trend was also observed for the other two configurations energetically close to AB_Mo/Zr_, see Table S2. Consistent with intuition, compressive strain reduces bond lengths while tensile strain increases them; see Table S3. However, in the ZrGe_2_N_4_ monolayer, the length of the Ge–N bond at the interface changes more significantly compared to the inner bonds of the same kind, whereas in MoS_2_, both Mo–S bonds change equally, see Table S3.

### 3.2 Electronic Properties of the Strain-free Heterostructure

**Figure 2:**
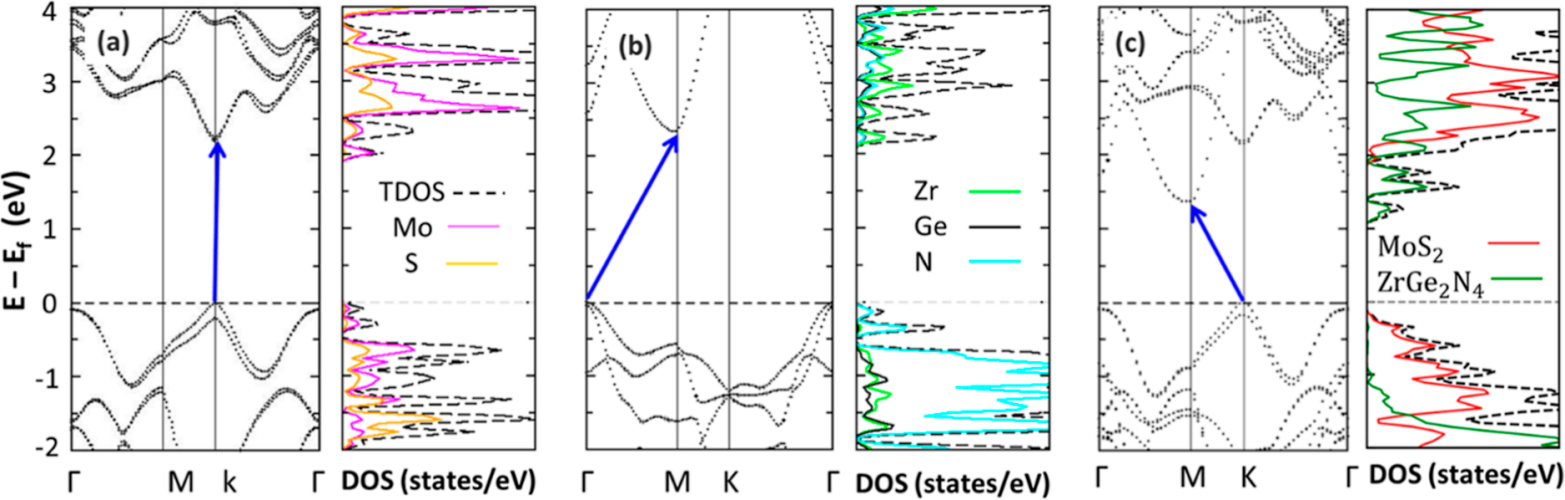
Electronic band structure and density of states of (a) monolayer MoS_2_, (b) monolayer ZrGe_2_N_4_, and (c) the heterostructure MoS_2_/ZrGe_2_N_4_ with stacking AB_Mo/Zr_, respectively, calculated with the HSE06 functional. The Fermi level is set to zero in all panels and marked by a horizontal dashed line. The blue arrows mark the fundamental gap.

To set a proper reference point for the analysis of the electronic properties of the MoS_2_/ZrGe_2_N_4_ HBL, it is instructive to start by examining its constituents. According to our HSE06 calculations, monolayer MoS_2_ features a direct band of 2.16 eV at K (Figure 2a) in agreement with the existing literature,^32,67^ while monolayer ZrGe_2_N_4_ has an indirect band gap of 2.34 eV between Γ and M. To the best of our knowledge, there is no report of the band gap of monolayer ZrGe_2_N_4_ computed with HSE06 but our result obtained with PBE (see Figure S3) agrees well with corresponding values from the literature.^54^ The direct band gap of ZrGe_2_N_4_ is at Γ, and it is 250 meV larger than the indirect one. SOC gives rise to a 210 meV splitting at the top of the valence band (VB) of MoS_2_.^31,32^ In contrast, no SOC splitting appears at any of the frontier states of ZrGe_2_N_4_.

The character of the electronic states can be inferred from the projected density of states (PDOS). In the case of MoS_2_, the highest occupied state exhibits hybridization between Mo and S atoms, while the lowest unoccupied state has a predominant Mo character, see Figure 2a. In the considered energy range, the contribution of Mo is always larger than that of the S atoms in the unoccupied region. In the valence, states with S character dominate below −1.0 eV, while equal contribution from Mo and S is found at lower energies.^68^ In ZrGe_2_N_4_, the highest occupied state originates solely from N atoms, whereas the lowest unoccupied state is a hybrid state with contributions from all elements with a predominance of Zr, see Figure 2b. The valence states of ZrGe_2_N_4_ have mainly N character.

The MoS_2_/ZrGe_2_N_4_ HBL in the AB_Mo/Zr_ stacking has an indirect band gap with the valence-band maximum (VBM) at K and the conduction band minimum (CBM) at M, see Figure 2c. A comparison with Figure 2a reveals that the top of the valence region is inherited from MoS_2_, with the VBM at K and a spin-orbit splitting of the highest occupied band of 159 meV slightly reduced compared to the isolated TMDC monolayer, see Figure 2a. Interestingly, the valence state at Γ becomes energetically closer to the VBM (77 meV) in the HBL compared to the isolated monolayer, as seen in TMDC heterostructures.^32,37^ On the other hand, the bottom of the conduction region is dominated by the features of ZrGe_2_N_4_ with the CBM at M as in the isolated system, while the lowest unoccupied state at the zone center remains 250 meV above CBM. The second unoccupied band originates from MoS_2_ where the valley at K remains clearly visible. These characteristics appear with even more clarity from the inspection of the PDOS. The type-II level alignment is evident, and so is the energy separation between the frontier states localized at opposite ends of the HBL. Deeper valence states and higher conduction states exhibit hybridization between the two constituents of the vdW-HS. As an example, a ZrGe_2_N_4_-related bulge appears immediately below the highest occupied states, Figure 2c.

### 3.3 Effects of Biaxial Strain

We continue our analysis by considering the MoS_2_/ZrGe_2_N_4_ HBL under strain. Due to the matching lattice constants of its building blocks, this interface is ideally suited to explore the effects of biaxial extensions and compressions equally distributed on both layers. Experimentally, this scenario can be realized with an appropriate choice of the substrate.^69−71^ In this study, we consider values of strain up to ±4% with intermediate steps at ±1 and ±2%. We chose this range as it is mostly explored in experiments on TMDCs.^72^ Higher values of strain can be realized under specific mechanical deformations which, however, typically induce curvature in the samples.^73−76^ Larger values of strain in flat TMDC monolayers have been studied from first principles^34^ to provide a point of reference for such extreme cases.

**Figure 3:**
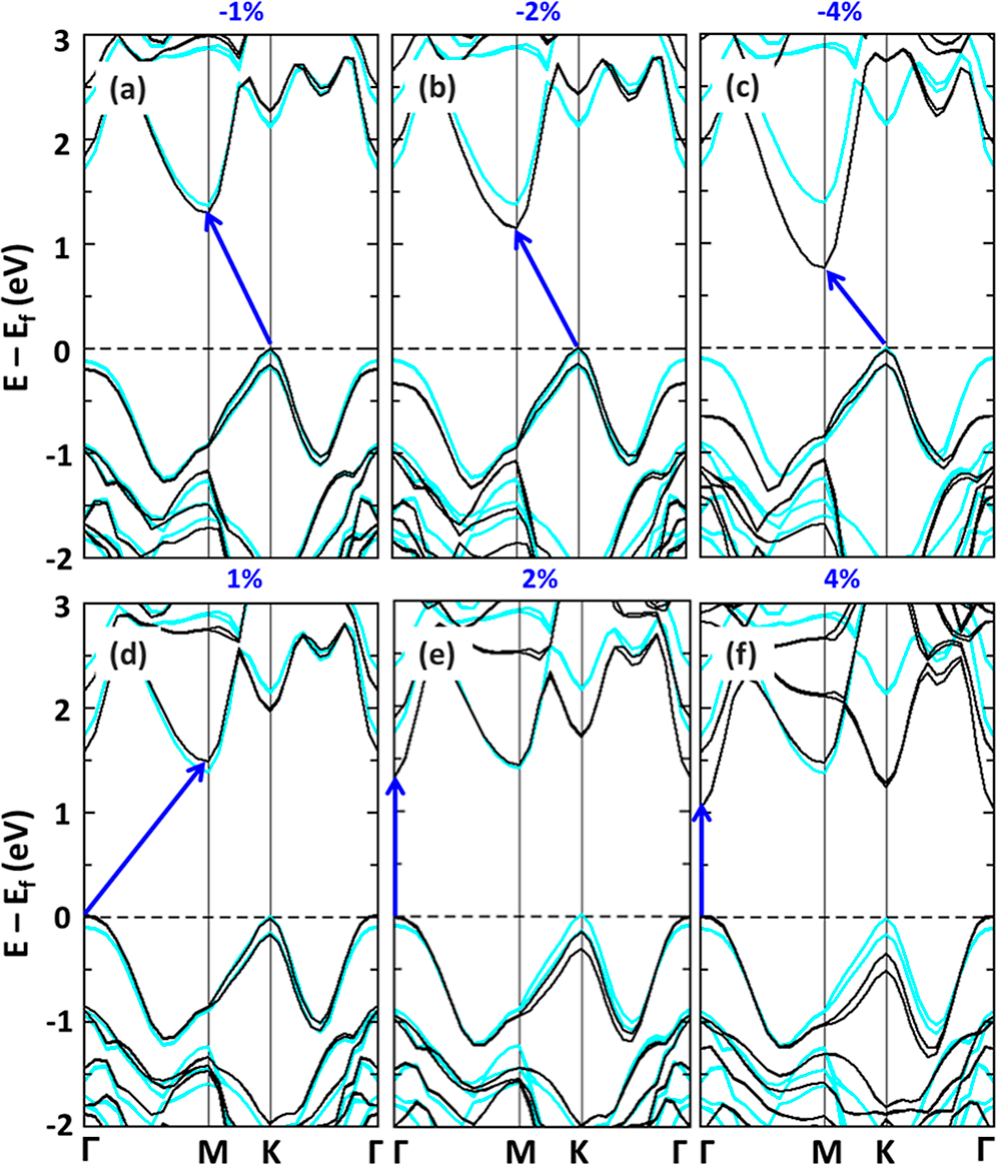
Electronic band structures of vdW AB_Mo/Zr_ HBL (black lines) under different values of compressive strain (a–c) and tensile strain (d–f) calculated using the HSE06 functional. The band structure of the unstrained HBL is shown for comparison (cyan lines) in each panel. The fundamental gap is marked by a blue arrow. The Fermi energy (*E*_f_) is set to zero at the VBM.

The band structures reported in Figure 3 indicate that the size of the fundamental gap reduces with increasing compressive and tensile strain, in analogy with to other 2D materials.^34,77−79^ However, the nature of the fundamental gap changes depending on the applied deformation. Under compressive strain (Figure 3a–c), the gap remains indirect with the VBM at K and the CBM at M. Direct comparison with the band structure of the unstrained HBL (cyan lines) reveals a significant downshift of the CBM which is responsible for the reduction of the gap upon increasing strain. Notably, in the valence region, the highest occupied states at Γ are found at lower energy compared with the VBM as strain is enhanced. Under tensile strain, the situation is more intricate. With 1% deformation, the fundamental band gap (blue arrow) is still indirect, but the direct band gap at Γ is only 88 meV larger (see Figure 3d). Further deformation (strain > 1%) is sufficient to downshift the lowest conduction state at Γ and to raise its counterpart in the valence region, giving rise to a direct band gap (Figure 3e). This trend is amplified, especially in the unoccupied region, upon 4% strain, under which the gap remains direct at Γ and shrinks further compared with the unstrained HBL (Figure 3f). We checked that these trends are independent of the stacking order, see Figure S6. As shown in Figure 3, strain primarily induces an indirect-to-direct band gap transition and shifts the valleys. No visible effect is produced on the band dispersion and thus on the corresponding effective masses (see Table S4).

**Figure 4:**
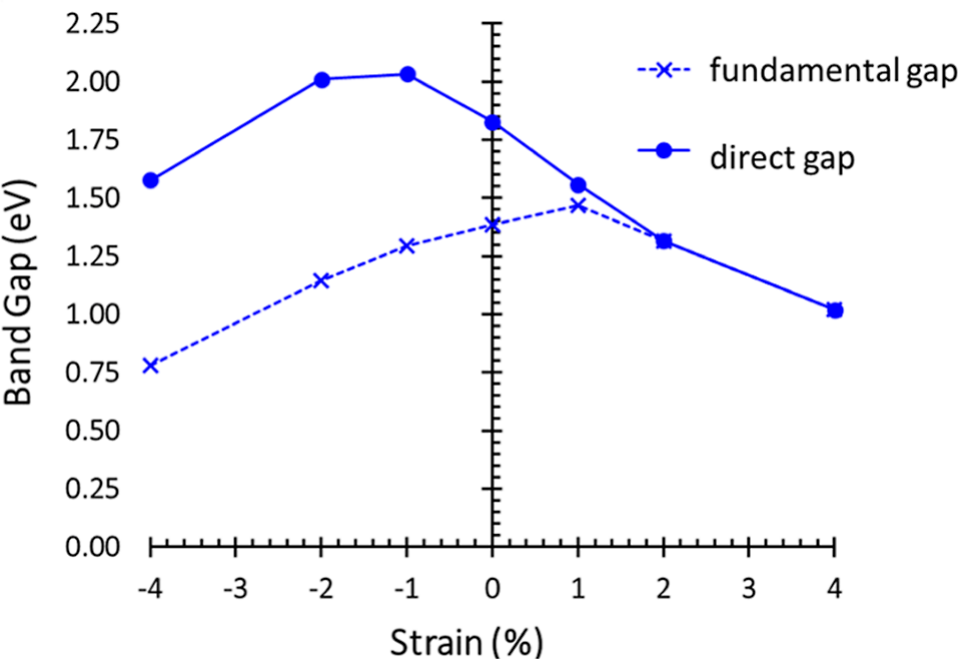
Fundamental (dotted lines) and direct (solid lines) band gaps of the MoS_2_/ZrGe2N_4_ HBL, calculated with the HSE06 functional including SOC, as a function of strain.

In Figure 4, we summarize the band-gap values of the HBL as a function of strain, distinguishing between fundamental and direct band gaps. At a glance, we notice that a large amount of tensile strain (≥2%) leads to an indirect-to-direct band gap transition but with band gap sizes smaller than in the unstrained case. Overall, the application of strain leads to a reduction of the fundamental gap, except for 1% strain. The direct band gap follows a completely different trend, being maximized under moderate compressive strain (−1%) and decreasing with tensile deformations. This behavior is generally reflected in the optical spectra computed in the independent-particle approximation on top of the HSE06 electronic structure, see Figure S8.

**Figure 5:**
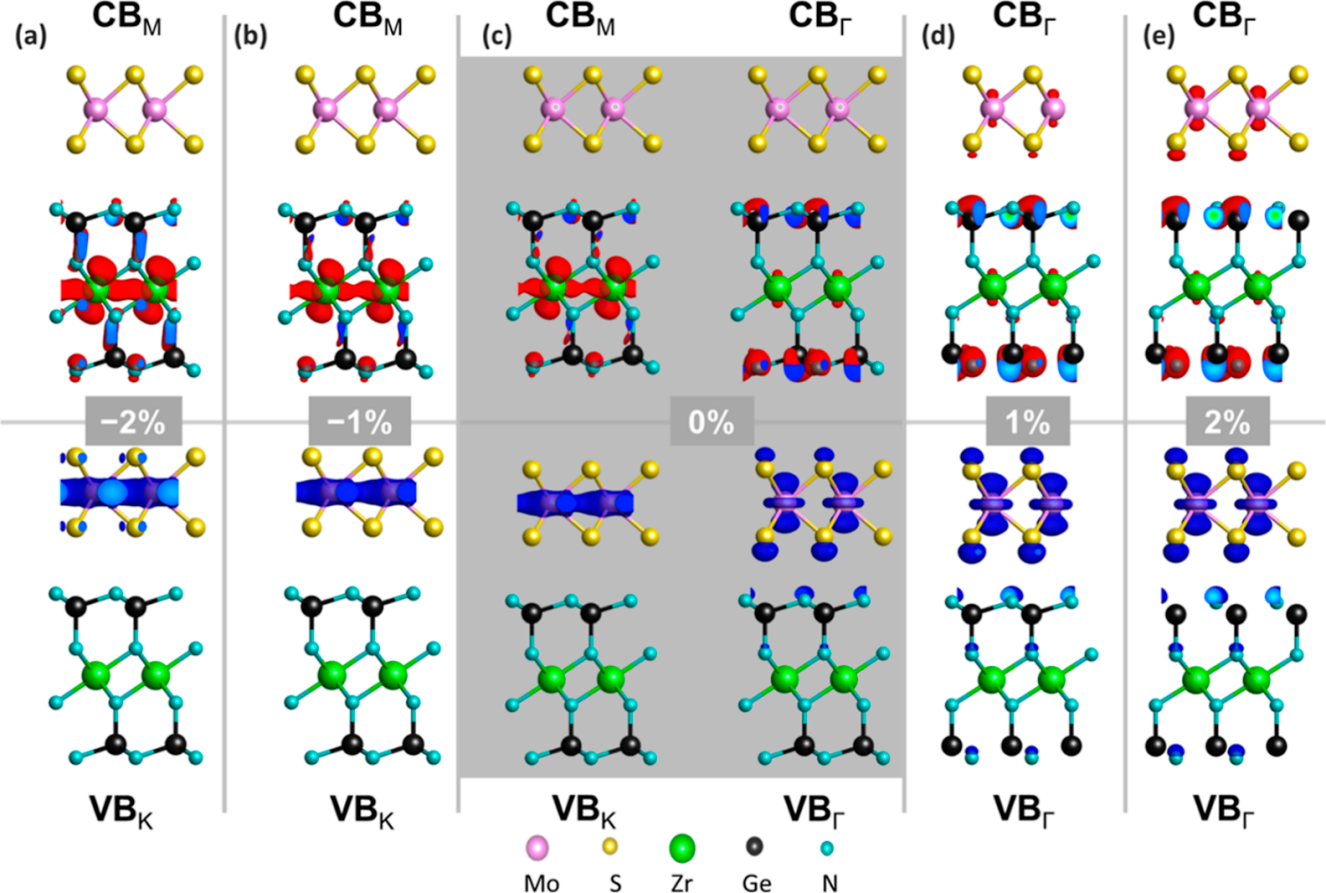
Real space visualization of the square modulus of the WFs of the MoS_2_/ZrGe_2_N_4_ HBL at the highest valence band (VB) and lowest conduction band (CB) at the high-symmetry points indicated in the subscript under (a) −2% and (b) −1% (compressive) strain, (c) without strain (0%), and under (d) 1% and (e) 2% (tensile) strain. The isovalue is set to 0.001 e/Å^3^.

To gain a deeper understanding of the consequences of strain on the electronic structure of the MoS_2_/ZrGe_2_N_4_ HBL and in particular on the reason for the indirect-to-direct band gap transition as a function of strain, we analyze the character of its frontier states by plotting in real space the square modulus of the corresponding wave functions (WFs), see Figure 5. In the unstrained configuration, the VBM at K is entirely localized on MoS_2_, while the CBM at M is solely on ZrGe_2_N_4_ and in particular around the Zr atoms, see Figure 5c. The distribution on the lowest conduction state at Γ remains localized on ZrGe_2_N_4_, although the character of the state changes and the probability density is maximized around the Ge–N bonds in the outermost layer. In contrast, in the valence region, the highest state at Γ is partially distributed also on ZrGe_2_N_4_ and in particular on the outermost layer of N atoms in close proximity with the outer distribution of the S p-orbitals in MoS_2_.

Under compressive strain, the WF probability remains localized on MoS_2_ in the valence region and on ZrGe_2_N_4_ in the conduction regardless of the applied amount (see Figure 5a,b). Visually, the two distributions are identical except for a small contribution from the S atoms in the highest occupied state at K. On the other hand, under tensile strain, a delocalization of the WF distribution is evident. This is especially remarkable in the conduction region, where the probability extends to MoS_2_. The larger contribution of MoS_2_ (ZrGe_2_N_4_) to the lowest unoccupied (highest occupied) state at the Γ-point is mirrored by an increase in the interlayer distance, see Table S2. This increased level of hybridization is also associated with the downshift of the lowest unoccupied valley at Γ, leading to an indirect-to-direct band gap transition.

### 3.4 Summary and Conclusions

In summary, we presented a comprehensive analysis of the electronic properties of HBL formed by monolayer MoS_2_ on top of the 1T-phase of ZrGe_2_N_4_. These materials have identical lattice parameters offering the opportunity to build a strain-free HBL. Among the considered stackings, the one in which the Mo atoms lie on top of the Zr atom is energetically most favorable, although differences in the total energies with most of the other configurations are on the order of a few meV. This HBL is characterized by an indirect band gap with the VBM and CBM at the high symmetry points K and M, respectively, and a type-II level alignment with the VBM (CBM) localized on MoS_2_ (ZrGe_2_N_4_). We analyzed the effects of biaxial strain on the electronic properties of HBL considering deformations up to ±4% of the in-plane lattice parameter at equilibrium. The size of the band gap reduces with increasing amounts of both tensile and compressive strain. Under tensile strain >1%, an indirect-to-direct transition occurs, shifting the CBM and VBM to the center of the Brillouin zone. The real-space analysis of the WF distribution confirms the localization of the highest occupied and lowest unoccupied states on the MoS_2_ and ZrGe_2_N_4_ monolayers, respectively. Yet, the VBM at Γ also includes a small contribution from the N atoms of ZrGe_2_N_4_ facing MoS_2_. Increasing tensile strain increases the amount of MoS_2_ character in the CBM as well as the contribution of the N atoms of ZrGe_2_N_4_ in the highest occupied states. Contrarily, compression only increases the concentration of each layer in the frontier states of HBL.

In conclusion, the vdW-HS MoS_2_/ZrGe_2_N_4_ subject to ∼2% of tensile strain with its direct band gap and type-II band alignment offers favorable perspectives for enhanced photoluminescent efficiency^80^ and ultrafast charge separation,^35,81−83^ thus covering a broad range of possible optoelectronic applications. Moreover, the direct band gap at Γ promises high solar efficiency in analogy with group III–V semiconductors (e.g., GaAs and InAs). Most importantly, the insight offered by this study suggests the potential of using strain to customize the electronic properties of vdW-HS in a controlled way. We finally emphasize the predictive power of *ab initio* simulations in discovering new material combinations such as MoS_2_/ZrGe_2_N_4_ with favorable structural characteristics such as absence of lattice mismatch. Further studies in this direction may extend the spectrum of available vdW-HS, starting, for example, by considering both polymorphs of MoS_2_ and ZrGe_2_N_4_ (1T and 2H phases) or using data-driven methods to identify other strain-free material combinations.

## Data Availability

The data that support the findings of this study are available on the Zenodo database with DOI: 10.5281/zenodo.10877727.

## Supporting Information Available

Structural analysis data, PBE band structures, phonon band structures, effective masses, and imaginary part of the dielectric function (PDF)

## References

[ref1] Schwierz F. Graphene Transistors. Nat. Nanotechnol. 2010, 5 (7), 487–496. 10.1038/nnano.2010.89. 20512128

[ref2] Wang Q. H.; Kalantar-Zadeh K.; Kis A.; Coleman J. N.; Strano M. S. Electronics and Optoelectronics of Two-Dimensional Transition Metal Dichalcogenides. Nat. Nanotechnol. 2012, 7 (11), 699–712. 10.1038/nnano.2012.193. 23132225

[ref3] Fiori G.; Bonaccorso F.; Iannaccone G.; Palacios T.; Neumaier D.; Seabaugh A.; Banerjee S. K.; Colombo L. Electronics Based on Two-Dimensional Materials. Nat. Nanotechnol. 2014, 9 (10), 768–779. 10.1038/nnano.2014.207. 25286272

[ref4] Liu Y.; Cheng R.; Liao et al L.; Liao L.; Zhou H.; Bai J.; Liu G.; Liu L.; Huang Y.; Duan X. Plasmon resonance enhanced multicolour photodetection by graphene. Nat. Commun. 2011, 2, 579 10.1038/ncomms1589. 22146398 PMC4235953

[ref5] Jariwala D.; Sangwan V. K.; Lauhon J. L.; Marks T. J.; Hersam M. Emerging Device Applications for Semiconducting Two-Dimensional Transition Metal Dichalcogenides. ACS Nano 2014, 8 (2), 1102 10.1021/nn500064s. 24476095

[ref6] Weiss N. O.; Zhou H.; Liao L.; Liu Y.; Jiang S.; Huang Y.; Duan X. Graphene: An Emerging Electronic Material. Adv. Mater. 2012, 24 (43), 5782–5825. 10.1002/adma.201201482. 22930422 PMC11524146

[ref7] Ramzan M. S.; Kuc A. B.; Kim H. S. Electronic Fingerprint Mechanism of NOx Sensor Based on Single-Material SnP3 Logical Junction. npj Comput. Mater. 2022, 8 (1), 220 10.1038/s41524-022-00903-7.

[ref8] Nag A.; Raidongia K.; Hembram K. P. S. S.; Datta R.; Waghmare U. V.; Rao C. N. R. Graphene Analogues of BN: Novel Synthesis and Properties. ACS Nano 2010, 4 (3), 1539–1544. 10.1021/nn9018762. 20128601

[ref9] Liu H.; Neal A. T.; Zhu Z.; Luo Z.; Xu X.; Tománek D.; Ye P. D. Phosphorene: An Unexplored 2D Semiconductor with a High Hole Mobility. ACS Nano 2014, 8 (4), 4033–4041. 10.1021/nn501226z. 24655084

[ref10] Qiao J.; Kong X.; Hu Z.-X.; Yang F.; Ji W. High-Mobility Transport Anisotropy and Linear Dichroism in Few-Layer Black Phosphorus. Nat. Commun. 2014, 5 (1), 4475 10.1038/ncomms5475. 25042376 PMC4109013

[ref11] Zhu Z.; Tománek D. Semiconducting Layered Blue Phosphorus: A Computational Study. Phys. Rev. Lett. 2014, 112 (17), 176802 10.1103/PhysRevLett.112.176802. 24836265

[ref12] Bhimanapati G. R.; Lin Z.; Meunier V.; Jung Y.; Cha J.; Das S.; Xiao D.; Son Y.; Strano M. S.; Cooper V. R.; Liang L.; Louie S. G.; Ringe E.; Zhou W.; Kim S. S.; Naik R. R.; Sumpter B. G.; Terrones H.; Xia F.; Wang Y.; Zhu J.; Akinwande D.; Alem N.; Schuller J. A.; Schaak R. E.; Terrones M.; Robinson J. A. Recent Advances in Two-Dimensional Materials beyond Graphene. ACS Nano 2015, 9 (12), 11509–11539. 10.1021/acsnano.5b05556. 26544756

[ref13] Heine T. Transition Metal Chalcogenides: Ultrathin Inorganic Materials with Tunable Electronic Properties. Acc. Chem. Res. 2015, 48 (1), 65–72. 10.1021/ar500277z. 25489917

[ref14] Lv R.; Terrones H.; Elías A. L.; Perea-López N.; Gutiérrez H. R.; Cruz-Silva E.; Rajukumar L. P.; Dresselhaus M. S.; Terrones M. Two-Dimensional Transition Metal Dichalcogenides: Clusters, Ribbons, Sheets and More. Nano Today 2015, 10 (5), 559–592. 10.1016/j.nantod.2015.07.004.

[ref15] Choi W.; Choudhary N.; Han G. H.; Park J.; Akinwande D.; Lee Y. H. Recent Development of Two-Dimensional Transition Metal Dichalcogenides and Their Applications. Mater. Today 2017, 20 (3), 116–130. 10.1016/j.mattod.2016.10.002.

[ref16] VahidMohammadi A.; Rosen J.; Gogotsi Y. The World of Two-Dimensional Carbides and Nitrides (MXenes). Science 2021, 372 (6547), eabf1581 10.1126/science.abf1581. 34112665

[ref17] Jing Y.; Ma Y.; Li Y.; Heine T. GeP3: A Small Indirect Band Gap 2D Crystal with High Carrier Mobility and Strong Interlayer Quantum Confinement. Nano Lett. 2017, 17 (3), 1833–1838. 10.1021/acs.nanolett.6b05143. 28125237

[ref18] Sun S.; Meng F.; Wang H.; Wang H.; Ni Y. Novel Two-Dimensional Semiconductor SnP 3: High Stability, Tunable Bandgaps and High Carrier Mobility Explored Using First-Principles Calculations. J. Mater. Chem. A 2018, 6 (25), 11890–11897. 10.1039/C8TA02494D.

[ref19] Ramzan M. S.; Bacic V.; Jing Y.; Kuc A. Electronic Properties of a New Family of Layered Materials from Groups 14 and 15: First-Principles Simulations. J. Phys. Chem. C 2019, 123 (41), 25470–25476. 10.1021/acs.jpcc.9b07068.

[ref20] Wang S.-S.; Sun W.; Dong S. Quantum Spin Hall Insulators and Topological Rashba-Splitting Edge States in Two-Dimensional CX3 (X = Sb, Bi). Phys. Chem. Chem. Phys. 2021, 23 (3), 2134–2140. 10.1039/D0CP05374K. 33437975

[ref21] Mak K. F.; Lee C.; Hone J.; Shan J.; Heinz T. F. Atomically Thin MoS_2: A New Direct-Gap Semiconductor. Phys. Rev. Lett. 2010, 105 (13), 136805 10.1103/PhysRevLett.105.136805. 21230799

[ref22] Radisavljevic B.; Radenovic A.; Brivio J.; Giacometti V.; Kis A. Single-Layer MoS2 Transistors. Nat. Nanotechnol. 2011, 6 (3), 147–150. 10.1038/nnano.2010.279. 21278752

[ref23] Qian X.; Liu J.; Fu L.; Li J. Quantum Spin Hall Effect in Two-Dimensional Transition Metal Dichalcogenides. Science 2014, 346 (6215), 1344–1347. 10.1126/science.1256815. 25504715

[ref24] Mak K. F.; Shan J. Photonics and Optoelectronics of 2D Semiconductor Transition Metal Dichalcogenides. Nat. Photonics 2016, 10 (4), 216–226. 10.1038/nphoton.2015.282.

[ref25] Mueller T.; Malic E. Exciton Physics and Device Application of Two-Dimensional Transition Metal Dichalcogenide Semiconductors. npj 2D Mater. Appl. 2018, 2 (1), 29 10.1038/s41699-018-0074-2.

[ref26] Gu G.; Xie Z. Modulation Doping of Graphene: An Approach toward Manufacturable Devices. Appl. Phys. Lett. 2011, 98 (8), 083502 10.1063/1.3556587.

[ref27] Geim A. K.; Grigorieva I. V. Van Der Waals Heterostructures. Nature 2013, 499 (7459), 419–425. 10.1038/nature12385. 23887427

[ref28] Gong Y.; Lin J.; Wang X.; Shi G.; Lei S.; Lin Z.; Zou X.; Ye G.; Vajtai R.; Yakobson B. I.; Terrones H.; Terrones M.; Tay B. K.; Lou J.; Pantelides S. T.; Liu Z.; Zhou W.; Ajayan P. M. Vertical and In-Plane Heterostructures from WS 2/MoS 2 Monolayers. Nat. Mater. 2014, 13 (12), 1135–1142. 10.1038/nmat4091. 25262094

[ref29] Novoselov K. S.; Mishchenko A.; Carvalho A.; Castro Neto A. H. 2D Materials and van Der Waals Heterostructures. Science 2016, 353 (6298), aac9439 10.1126/science.aac9439. 27471306

[ref30] Blundo E.; Felici M.; Yildirim T.; Pettinari G.; Tedeschi D.; Miriametro A.; Liu B.; Ma W.; Lu Y.; Polimeni A. Evidence of the Direct-to-Indirect Band Gap Transition in Strained Two-Dimensional WS 2, MoS 2, and WSe 2. Phys. Rev. Research 2020, 2 (1), 012024 10.1103/PhysRevResearch.2.012024.

[ref31] Krumland J.; Cocchi C. Conditions for Electronic Hybridization between Transition-Metal Dichalcogenide Monolayers and Physisorbed Carbon-Conjugated Molecules. Electron. Struct. 2021, 3 (4), 044003 10.1088/2516-1075/ac421f.

[ref32] Ramzan M. S.; Kunstmann J.; Kuc A. B. Tuning Valleys and Wave Functions of van Der Waals Heterostructures by Varying the Number of Layers: A First-Principles Study. Small 2021, 17, 2008153 10.1002/smll.202008153. 33955665

[ref33] Ramzan M. S.; Goodwin Z. A. H.; Mostofi A. A.; Kuc A.; Lischner J. Effect of Coulomb Impurities on the Electronic Structure of Magic Angle Twisted Bilayer Graphene. npj 2D Mater. Appl. 2023, 7 (1), 49 10.1038/s41699-023-00403-2.

[ref34] Ramzan M. S.; Cocchi C. Strained Monolayer MoTe2 as a Photon Absorber in the Telecom Range. Nanomaterials 2023, 13 (20), 2740 10.3390/nano13202740. 37887890 PMC10608843

[ref35] Hong X.; Kim J.; Shi S. F.; Zhang Y.; Jin C.; Sun Y.; Tongay S.; Wu J.; Zhang Y.; Wang F. Ultrafast Charge Transfer in Atomically Thin MoS2/WS2 Heterostructures. Nat. Nanotechnol. 2014, 9 (9), 682–686. 10.1038/nnano.2014.167. 25150718

[ref36] Fan S.; Vu Q. A.; Lee S.; Phan T. L.; Han G.; Kim Y.-M.; Yu W. J.; Lee Y. H. Tunable Negative Differential Resistance in van Der Waals Heterostructures at Room Temperature by Tailoring the Interface. ACS Nano 2019, 13 (7), 8193–8201. 10.1021/acsnano.9b03342. 31260265

[ref37] Kunstmann J.; Mooshammer F.; Nagler P.; Chaves A.; Stein F.; Paradiso N.; Plechinger G.; Strunk C.; Schüller C.; Seifert G.; Reichman D. R.; Korn T. Momentum-Space Indirect Interlayer Excitons in Transition Metal Dichalcogenide van Der Waals Heterostructures. Nat. Phys. 2018, 14 (8), 801–805. 10.1038/s41567-018-0123-y.

[ref38] Tang Y.; Li L.; Li T.; Xu Y.; Liu S.; Barmak K.; Watanabe K.; Taniguchi T.; MacDonald A. H.; Shan J.; Mak K. F. Simulation of Hubbard Model Physics in WSe2/WS2Moiré Superlattices. Nature 2020, 579 (7799), 353–358. 10.1038/s41586-020-2085-3. 32188950

[ref39] Regan E. C.; Wang D.; Jin C.; Bakti Utama M. I.; Gao B.; Wei X.; Zhao S.; Zhao W.; Zhang Z.; Yumigeta K.; Blei M.; Carlström J. D.; Watanabe K.; Taniguchi T.; Tongay S.; Crommie M.; Zettl A.; Wang F. Mott and Generalized Wigner Crystal States in WSe2/WS2Moiré Superlattices. Nature 2020, 579 (7799), 359–363. 10.1038/s41586-020-2092-4. 32188951

[ref40] Wang L.; Shih E.-M.; Ghiotto A.; Xian L.; Rhodes D. A.; Tan C.; Claassen M.; Kennes D. M.; Bai Y.; Kim B.; Watanabe K.; Taniguchi T.; Zhu X.; Hone J.; Rubio A.; Pasupathy A. N.; Dean C. R. Correlated Electronic Phases in Twisted Bilayer Transition Metal Dichalcogenides. Nat. Mater. 2020, 19 (8), 861–866. 10.1038/s41563-020-0708-6. 32572205

[ref41] Li H.; Li S.; Naik M. H.; Xie J.; Li X.; Wang J.; Regan E.; Wang D.; Zhao W.; Zhao S.; Kahn S.; Yumigeta K.; Blei M.; Taniguchi T.; Watanabe K.; Tongay S.; Zettl A.; Louie S. G.; Wang F.; Crommie M. F. Imaging Moiré Flat Bands in Three-Dimensional Reconstructed WSe2/WS2 Superlattices. Nat. Mater. 2021, 20 (7), 945–950. 10.1038/s41563-021-00923-6. 33558718

[ref42] Hong Y.-L.; Liu Z.; Wang L.; Zhou T.; Ma W.; Xu C.; Feng S.; Chen L.; Chen M.-L.; Sun D.-M.; Chen X.-Q.; Cheng H.-M.; Ren W. Chemical Vapor Deposition of Layered Two-Dimensional MoSi2N4Materials. Science 2020, 369 (6504), 670–674. 10.1126/science.abb7023. 32764066

[ref43] Ai H.; Liu D.; Geng J.; Wang S.; Lo K. H.; Pan H. Theoretical evidence of the spin–valley coupling and valley polarization in two-dimensional MoSi_2_X_4_ (X = N, P, and As). Phys. Chem. Chem. Phys. 2021, 23 (4), 3144–3151. 10.1039/D0CP05926A. 33496290

[ref44] Mortazavi B.; Javvaji B.; Shojaei F.; Rabczuk T.; Shapeev A. V.; Zhuang X. Exceptional piezoelectricity, high thermal conductivity and stiffness and promising photocatalysis in two-dimensional MoSi2N4 family confirmed by first-principles. Nano Energy 2021, 82, 105716 10.1016/j.nanoen.2020.105716.

[ref45] Yao H.; Zhang C.; Wang Q.; Li J.; Yu Y.; Xu F.; Wang B.; Wei Y. Novel Two-Dimensional Layered MoSi2Z4 (Z = P, As): New Promising Optoelectronic Materials. Nanomaterials 2021, 11 (3), 559 10.3390/nano11030559. 33668165 PMC7995989

[ref46] Woźniak T.; Umm-e hani.; Faria Junior P. E.; Ramzan M. S.; Kuc A. B. Electronic and Excitonic Properties of MSi2Z4Monolayers. Small 2023, 19 (19), 2206444 10.1002/smll.202206444. 36772899

[ref47] Yin Y.; Gong Q.; Yi M.; Guo W. Emerging Versatile Two-Dimensional MoSi2N4 Family. Adv. Funct. Mater. 2023, 33 (26), 2214050 10.1002/adfm.202214050.

[ref48] Ramzan M. S.; Woźniak T.; Kuc A.; Cocchi C. Composition-Dependent Absorption of Radiation in Semiconducting MSi _2_ Z _4_ Monolayers. Phys. Status Solidi B 2024, 261, 2300570 10.1002/pssb.202300570.

[ref49] Wang J.; Shu H.; Liang P.; Wang N.; Cao D.; Chen X. Thickness-Dependent Phase Stability and Electronic Properties of GaN Nanosheets and MoS2/GaN van Der Waals Heterostructures. J. Phys. Chem. C 2019, 123 (6), 3861–3867. 10.1021/acs.jpcc.8b10915.

[ref50] Yang F.; Han J.; Zhang L.; Tang X.; Zhuo Z.; Tao Y.; Cao X.; Dai Y. Adjustable Electronic and Optical Properties of BlueP/MoS2 van Der Waals Heterostructure by External Strain: A First-Principles Study. Nanotechnology 2020, 31 (37), 375706 10.1088/1361-6528/ab978b. 32464615

[ref51] Bafekry A.; Faraji M.; Abdollahzadeh Ziabari A.; Fadlallah M. M.; Nguyen C. V.; Ghergherehchi M.; Feghhi S. a. H. A van Der Waals Heterostructure of MoS2/MoSi2N4: A First-Principles Study. New J. Chem. 2021, 45 (18), 8291–8296. 10.1039/D1NJ00344E.

[ref52] Cao L.; Zhou G.; Wang Q.; Ang L. K.; Ang Y. S. Two-Dimensional van Der Waals Electrical Contact to Monolayer MoSi2N4. Appl. Phys. Lett. 2021, 118 (1), 013106 10.1063/5.0033241.

[ref53] Xu X.; Yang L.; Gao Q.; Jiang X.; Li D.; Cui B.; Liu D. Type-II MoSi2N4/MoS2 van Der Waals Heterostructure with Excellent Optoelectronic Performance and Tunable Electronic Properties. J. Phys. Chem. C 2023, 127 (16), 7878–7886. 10.1021/acs.jpcc.3c00773.

[ref54] Liu W.; Xie Y.; Yuan J.; Chen Y. Super High-Performance 7-Atomic-Layer Thermoelectric Material ZrGe2N4. Nanoscale 2022, 14 (24), 8797–8805. 10.1039/D2NR01848A. 35678526

[ref55] Voiry D.; Mohite A.; Chhowalla M. Phase Engineering of Transition Metal Dichalcogenides. Chem. Soc. Rev. 2015, 44 (9), 2702–2712. 10.1039/C5CS00151J. 25891172

[ref56] Wang L.; Shi Y.; Liu M.; Zhang A.; Hong Y.-L.; Li R.; Gao Q.; Chen M.; Ren W.; Cheng H.-M.; Li Y.; Chen X.-Q. Intercalated Architecture of MA2Z4 Family Layered van Der Waals Materials with Emerging Topological, Magnetic and Superconducting Properties. Nat. Commun. 2021, 12 (1), 2361 10.1038/s41467-021-22324-8. 33883547 PMC8060390

[ref57] Hohenberg P.; Kohn W. Inhomogeneous Electron Gas. Phys. Rev. 1964, 136 (3B), B864–B871. 10.1103/PhysRev.136.B864.

[ref58] Kresse G.; Furthmüller J. Efficient Iterative Schemes for *Ab Initio* Total-Energy Calculations Using a Plane-Wave Basis Set. Phys. Rev. B 1996, 54 (16), 11169–11186. 10.1103/PhysRevB.54.11169. 9984901

[ref59] Blöchl P. E. Projector Augmented-Wave Method. Phys. Rev. B 1994, 50 (24), 17953–17979. 10.1103/PhysRevB.50.17953. 9976227

[ref60] Perdew J. P.; Burke K.; Ernzerhof M. Generalized Gradient Approximation Made Simple. Phys. Rev. Lett. 1996, 77 (18), 3865–3868. 10.1103/PhysRevLett.77.3865. 10062328

[ref61] Grimme S.; Antony J.; Ehrlich S.; Krieg H. A Consistent and Accurate Ab Initio Parametrization of Density Functional Dispersion Correction (DFT-D) for the 94 Elements H-Pu. J. Chem. Phys. 2010, 132 (15), 154104 10.1063/1.3382344. 20423165

[ref62] Heyd J.; Scuseria G. E.; Ernzerhof M. Hybrid Functionals Based on a Screened Coulomb Potential. J. Chem. Phys. 2003, 118 (18), 8207–8215. 10.1063/1.1564060.

[ref63] Momma K.; Izumi F. VESTA 3 for Three-Dimensional Visualization of Crystal, Volumetric and Morphology Data. J. Appl. Crystallogr. 2011, 44 (6), 1272–1276. 10.1107/S0021889811038970.

[ref64] Kuc A.; Heine T. The Electronic Structure Calculations of Two-Dimensional Transition-Metal Dichalcogenides in the Presence of External Electric and Magnetic Fields. Chem. Soc. Rev. 2015, 44 (9), 2603–2614. 10.1039/C4CS00276H. 25529067

[ref65] Johari P.; Shenoy V. B. Tuning the Electronic Properties of Semiconducting Transition Metal Dichalcogenides by Applying Mechanical Strains. ACS Nano 2012, 6 (6), 5449–5456. 10.1021/nn301320r. 22591011

[ref66] Fitri D. A.; Purqon A. Calculation Study of Electric Properties on Molybdenum Disulfide By Using Density Functional Theory. J. Phys.: Conf. Ser. 2017, 877, 012071 10.1088/1742-6596/877/1/012071.

[ref67] Krumland J.; Cocchi C. Electronic Structure of Low-Dimensional Inorganic/Organic Interfaces: Hybrid Density Functional Theory, G 0 W 0, and Electrostatic Models. Phys. Status Solidi A 2024, 221 (1), 2300089 10.1002/pssa.202300089.

[ref68] Cao D.; Shu H. B.; Wu T. Q.; Jiang Z. T.; Jiao Z. W.; Cai M. Q.; Hu W. Y. First-Principles Study of the Origin of Magnetism Induced by Intrinsic Defects in Monolayer MoS2. Appl. Surf. Sci. 2016, 361, 199–205. 10.1016/j.apsusc.2015.11.134.

[ref69] Ahn G. H.; Amani M.; Rasool H.; Lien D.-H.; Mastandrea J. P.; Ager III J. W.; Dubey M.; Chrzan D. C.; Minor A. M.; Javey A. Strain-Engineered Growth of Two-Dimensional Materials. Nat. Commun. 2017, 8 (1), 608 10.1038/s41467-017-00516-5. 28931806 PMC5606995

[ref70] Wan Y.; Huang J.-K.; Chuu C.-P.; Hsu W.-T.; Lee C.-J.; Aljarb A.; Huang C.-W.; Chiu M.-H.; Tang H.-L.; Lin C.; Zhang X.; Wei C.-M.; Li S.; Chang W.-H.; Li L.-J.; Tung V. Strain-Directed Layer-By-Layer Epitaxy Toward van Der Waals Homo- and Heterostructures. ACS mater. lett 2021, 3 (4), 442–453. 10.1021/acsmaterialslett.0c00554.

[ref71] Ambos B.; Ramzan M. S.; Cocchi C.; Nilius N. Growth of Dichalcogenide Layers on TiO2(110)—MoSe2 or TiSe2. Phys. Status Solidi A 2023, 220 (21), 2300365 10.1002/pssa.202300365.

[ref72] Blundo E.; Di Giorgio C.; Pettinari G.; Yildirim T.; Felici M.; Lu Y.; Bobba F.; Polimeni A. Engineered Creation of Periodic Giant, Nonuniform Strains in MoS2Monolayers. Adv. Mater. Interfaces 2020, 7 (17), 2000621 10.1002/admi.202000621.

[ref73] Darlington T. P.; Carmesin C.; Florian M.; Yanev E.; Ajayi O.; Ardelean J.; Rhodes D. A.; Ghiotto A.; Krayev A.; Watanabe K.; Taniguchi T.; Kysar J. W.; Pasupathy A. N.; Hone J. C.; Jahnke F.; Borys N. J.; Schuck P. J. Imaging Strain-Localized Excitons in Nanoscale Bubbles of Monolayer WSe2 at Room Temperature. Nat. Nanotechnol. 2020, 15 (10), 854–860. 10.1038/s41565-020-0730-5. 32661371

[ref74] Blundo E.; Yildirim T.; Pettinari G.; Polimeni A. Experimental Adhesion Energy in van Der Waals Crystals and Heterostructures from Atomically Thin Bubbles. Phys. Rev. Lett. 2021, 127 (4), 046101 10.1103/PhysRevLett.127.046101. 34355951

[ref75] Krumland J.; Velja S.; Cocchi C. Quantum Dots in Transition Metal Dichalcogenides Induced by Atomic-Scale Deformations. ACS Photonics 2024, 11 (2), 586–595. 10.1021/acsphotonics.3c01470. 38405397 PMC10885200

[ref76] Velja S.; Krumland J.; Cocchi C. Electronic Properties of MoSe _2_ Nanowrinkles. Nanoscale 2024, 16 (14), 7134–7144. 10.1039/D3NR06261A. 38501908

[ref77] Conley H. J.; Wang B.; Ziegler J. I.; Haglund R. F.; Pantelides S. T.; Bolotin K. I. Bandgap Engineering of Strained Monolayer and Bilayer MoS _2_. Nano Lett. 2013, 13 (8), 3626–3630. 10.1021/nl4014748. 23819588

[ref78] Thomas S.; Manju M. S.; Ajith K. M.; Lee S. U.; Asle Zaeem M. Strain-Induced Work Function in h-BN and BCN Monolayers. Phys. E (Amsterdam, Neth.) 2020, 123, 114180 10.1016/j.physe.2020.114180.

[ref79] Chaves A.; Azadani J. G.; Alsalman H.; da Costa D. R.; Frisenda R.; Chaves A. J.; Song S. H.; Kim Y. D.; He D.; Zhou J.; Castellanos-Gomez A.; Peeters F. M.; Liu Z.; Hinkle C. L.; Oh S.-H.; Ye P. D.; Koester S. J.; Lee Y. H.; Avouris P.; Wang X.; Low T. Bandgap Engineering of Two-Dimensional Semiconductor Materials. npj 2D Mater. Appl. 2020, 4 (1), 29 10.1038/s41699-020-00162-4.

[ref80] Yu Y.; Dong C.-D.; Binder R.; Schumacher S.; Ning C.-Z. Strain-Induced Indirect-to-Direct Bandgap Transition, Photoluminescence Enhancement, and Linewidth Reduction in Bilayer MoTe2. ACS Nano 2023, 17 (5), 4230–4238. 10.1021/acsnano.2c01665. 36812007

[ref81] Zhang J.; Hong H.; Lian C.; Ma W.; Xu X.; Zhou X.; Fu H.; Liu K.; Meng S. Interlayer-State-Coupling Dependent Ultrafast Charge Transfer in MoS2/WS2 Bilayers. Advanced Science 2017, 4 (9), 1700086 10.1002/advs.201700086. 28932669 PMC5604380

[ref82] Miller B.; Steinhoff A.; Pano B.; Klein J.; Jahnke F.; Holleitner A.; Wurstbauer U. Long-Lived Direct and Indirect Interlayer Excitons in van Der Waals Heterostructures. Nano Lett. 2017, 17 (9), 5229–5237. 10.1021/acs.nanolett.7b01304. 28742367

[ref83] Jin C.; Ma E. Y.; Karni O.; Regan E. C.; Wang F.; Heinz T. F. Ultrafast Dynamics in van Der Waals Heterostructures. Nat. Nanotechnol. 2018, 13 (11), 994–1003. 10.1038/s41565-018-0298-5. 30397296

