# Supplementary material for: Strain-free MoS2/ZrGe2N4 van der Waals Heterostructure: Tunable Electronic Properties with Type-II Band Alignment
Source: ACS Omega. 2024 Jul 5;9(28):30717–24. doi: 10.1021/acsomega.4c03193 (PMC11256293; doi:10.1021/acsomega.4c03193)
Supplement: Supplementary file 1 — ao4c03193_si_001.pdf [file ao4c03193_si_001.pdf]

SUPPORTING INFORMATION

Strain-free MoS_2/$ZrGe_2N_4$ van der Waals Heterostructure: Tunable electronic properties with type-II band alignment

Mustapha Driouech[1†], Amrita Mitra[1, †,×], Caterina Cocchi[1,2*], Muhammad Sufyan Ramzan[1*]

[1]Institut für Physik, Carl von Ossietzky Universität, 26129 Oldenburg, Germany.

[2]Center for Nanoscale Dynamics (CeNaD), Carl von Ossietzky Universität, 26129, Oldenburg, Germany.

[†]These authors contributed equally.

[×]Present address: Okinawa Institute of Science and Technology, 1919-1 Tancha, Onna, Kunigami District, Okinawa 904-0412, Japan

Correspondence to:

muhammad.sufyan.ramzan@uni-oldenburg.de

caterina.cocchi@uni-oldenburg.de

a. Structural Properties

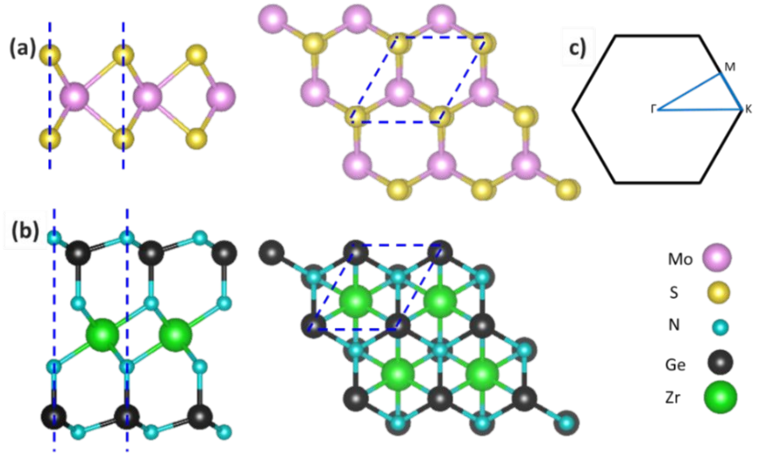

Figure S1 Ball and stick representation of monolayer a) MoS_2 and b) $ZrGe_2N_4$ with the side view shown on the left and the top view on the right; c) Brillouin zone (BZ) of both materials with the high symmetry points and the irreducible BZ formed by them marked by a blue triangle.

Table S1. Interlayer distance (d) and bond length ($d_{x\text{-}y}$, where x, y refers to different constituent elements) of the considered stacking configurations of $MoS_2/ZrGe_2N_4$ HBL. The results obtained for the energetically the most stable system is highlighted in bold and placed on a gray background. For reference, bond lengths of the isolated monolayers are also provided.

System	d (Å)	$d_{S\text{-}Mo}$ (Å)	$d_{Mo\text{-}S\_interface}$ (Å)	$d_{N\text{-}Ge}$ (Å)	$d_{Ge\text{-}N}$ (Å)	$d_{N\text{-}Zr}$ (Å)
$AA_{Mo/Ge}$	3.38	2.41	2.41	1.91	1.87	2.18
$AA_{Mo/Zr}$	3.36	2.41	2.40	1.91	1.87	2.18
$AB_{S/Ge}$	2.97	2.41	2.40	1.91	1.87	2.18
**$AB_{Mo/Zr}$**	**2.97**	**2.41**	**2.40**	**1.91**	**1.87**	**2.18**
$AB_{Mo/Ge}$	2.97	2.41	2.40	1.92	1.87	2.18
$AB_{Mo/N}$	2.97	2.41	2.40	1.91	1.87	2.18
AC	3.03	2.41	2.40	1.91	1.87	2.18
AC'	3.03	2.41	2.40	1.91	1.87	2.18
MoS_2	--	2.41	2.41	--	--	--
$ZrGe_2N_4$	--	--	--	1.91	1.87	2.18

Table S2. Interlayer distances for the three most stable configurations of the $MoS_2/ZrGe_2N_4$ HBL under the indicated values of compressive (negative) and tensile (positive) biaxial strain ε. The results obtained for the energetically the most stable system is highlighted in bold and placed on a gray background.

Systems	Interlayer distance (Å)						
	ε=-4%	ε=-2%	ε=-1%	ε=0%	ε=+1%	ε=+2%	ε=+4%
**$AB_{Mo/Zr}$**	**3.02**	**2.97**	**2.95**	**2.97**	**2.99**	**3.01**	**2.82**
AC	3.02	3.03	--	3.03	--	3.01	2.81
AC’	3.02	2.97	--	3.03	--	3.01	2.81

Table S3. Mo-S and Ge-N bond lengths in the $MoS_2/ZrGe_2N_4$ HBL with $AB_{S/Ge}$ stacking under different values of compressive (negative) and tensile (positive) biaxial strain ε. Bonds at the interface with the other materials are marked with subscript “interface”.

Bond length (Å)	strain (ε)						
	-4%	-2%	-1%	0	+1%	+2%	+4%
$d_{Mo\text{-}S}$	2.38	2.39	2.40	2.41	2.42	2.42	2.44
$d_{Mo\text{-}S\_interface}$	2.38	2.39	2.40	2.40	2.41	2.42	2.43
$d_{Ge\text{-}N\_interface}$	1.86	1.89	1.90	1.91	1.93	1.94	1.98
$d_{Ge\text{-}N}$	1.86	1.86	1.87	1.87	1.87	1.88	1.88

b. Electronic properties

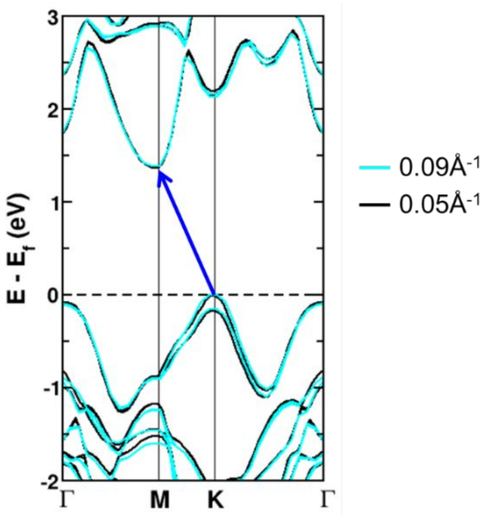

Figure S2. Band structure of the unstrained $MoS_2/ZrGe_2N_4$ HBL calculated with HSE06 using two different k-point meshes indicated in the legend by the spacing between neighboring k-points. The Fermi level is set to zero at the top of the valence band. The blue arrow marks the fundamental gap, which is unchanged with respect to the chosen k-mesh.

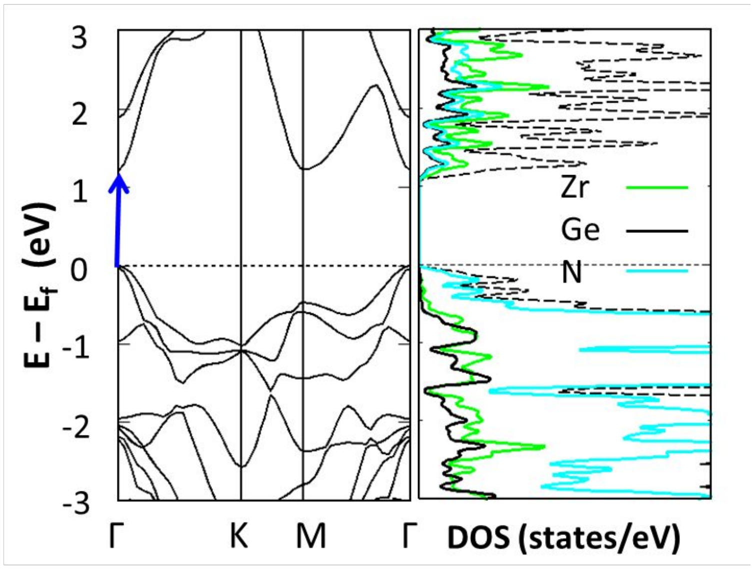

Figure S3. Electronic band structure (left) and atom-projected density of states (PDOS, right) of monolayer $ZrGe_2N_4$ calculated with PBE. The Fermi energy (E_f) is set to zero at the top of the valance band and the fundamental gap is marked by a blue arrow. In the PDOS, the sum of all atomic contributions (solid lines) is given by the dashed curve.

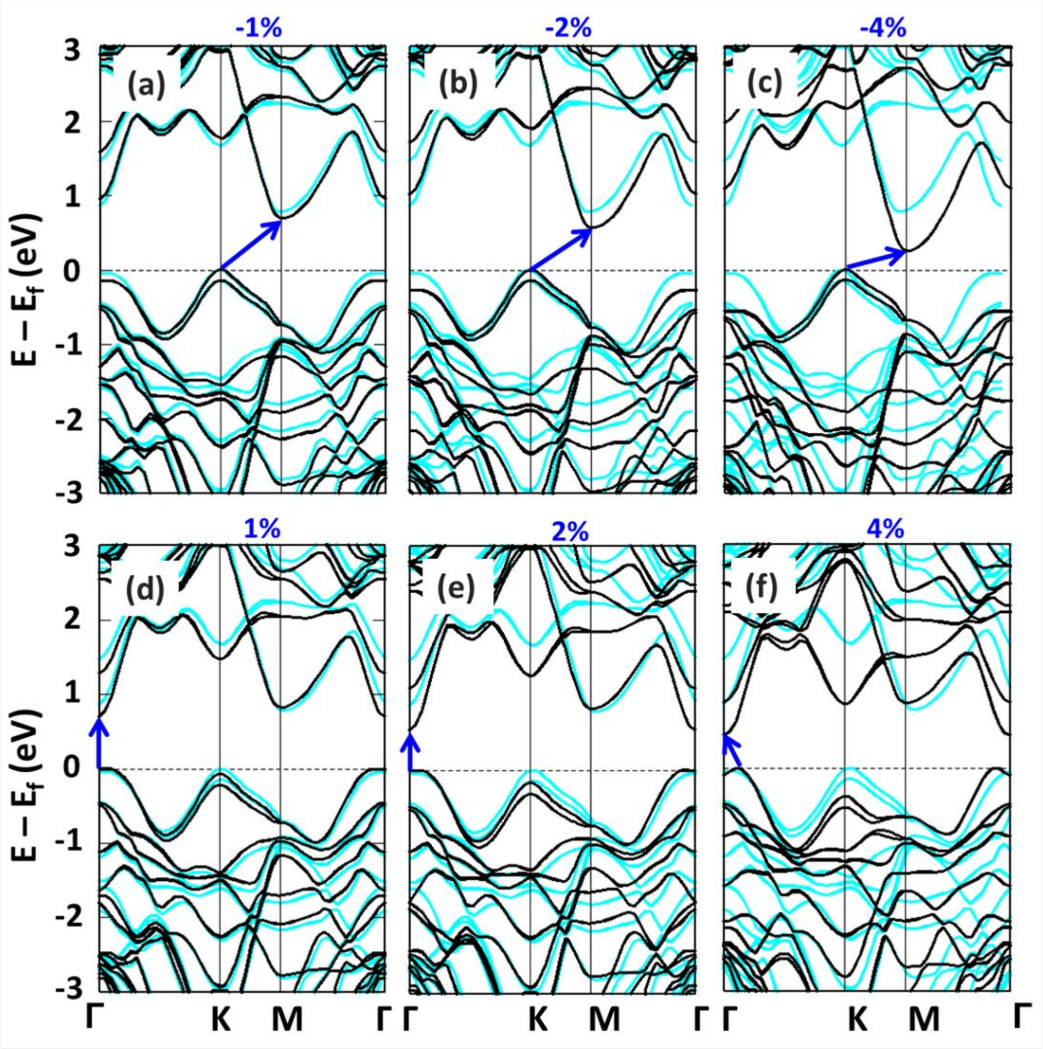

Figure S4. Electronic band structures of the $MoS_2/ZrGe_2N_4$ HBL ($AB_{Mo/Zr}$ stacking) calculated from PBE under different values of compressive (a - c) and tensile strain (d - f). The band structure of the unstrained HBL is shown in cyan for reference. The Fermi energy (E_f) is set to zero at the top of the valance band. The fundamental gap is marked by a blue arrow.

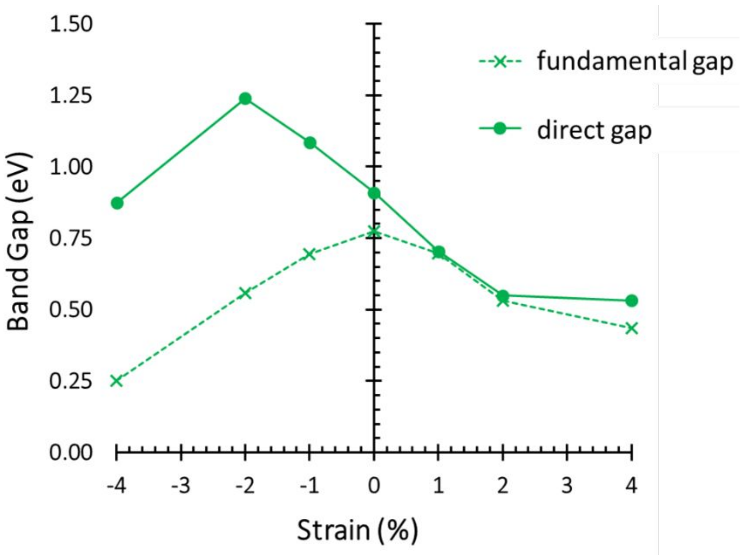

Figure S5. Fundamental and direct band gaps of the $MoS_2/ZrGe_2N_4$ HBL ($AB_{Mo/Zr}$ stacking) calculated from PBE and spin-orbit coupling as a function of strain.

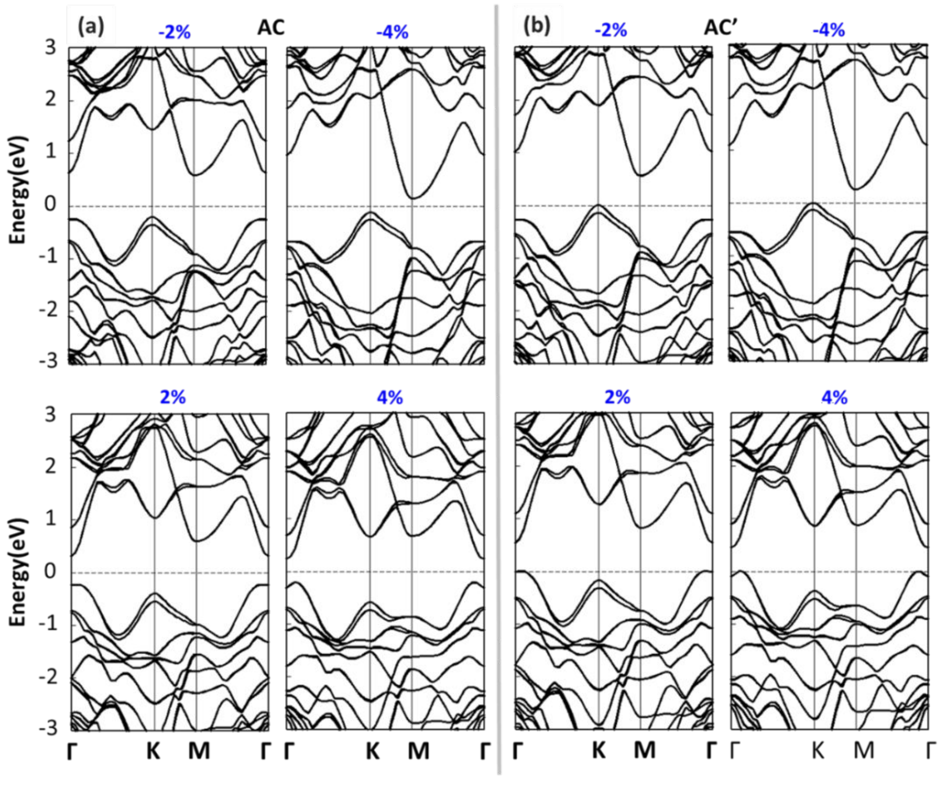

Figure S6. Electronic band structure of the $MoS_2/ZrGe_2N_4$ HBL in the a) AC and b) AC' stacking configuration under different values of compressive (top) and tensile strain (bottom).

Table S4. Effective masses in units of the free electron m_e for electrons and holes calculated for different values of strain at the top of the valence band (VBM) and at the bottom of the conduction band (CBM) at the high symmetry points indicated in the subscripts (see **Figure 3** of the main text).

	**$VBM_Γ$**			**VBM_K**				**CBM_M**					**$CBM_Γ$**	
**strain (ε)**	**1%**	**2%**	**4%**	**0%**	**-1%**	**-2%**	**-4%**	**0%**	**1%**	**-1%**	**-2%**	**-4%**	**2%**	**4%**
**m^*_h (m_e)**	0.71	0.79	0.63	0.69	0.64	0.68	0.71	-	-	-	-	-	-	-
**m^*_e (m_e)**	-	-	-	-	-	-	-	0.50	0.71	0.49	0.48	0.47	0.35	0.33

c. Vibrational properties

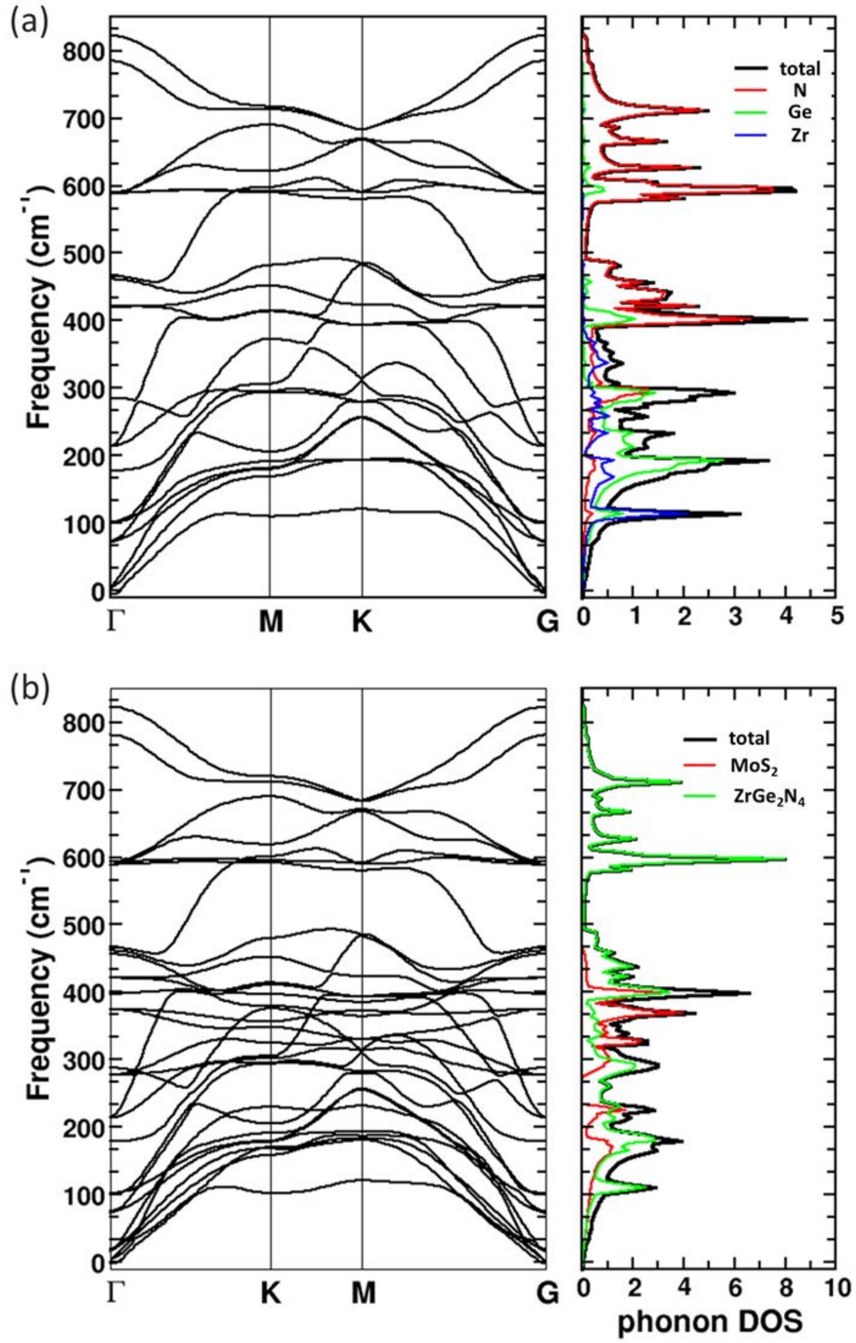

Figure S7 Phonon band structure and projected density of states of (a) $ZrGe_2N_4$ monolayer and (b) $ZrGe_2N_4/MoS_2$ HBL computed using the finite displacement method, as implemented in Phonopy, in a 3×3×1 supercell of the HBL.

d. Optical properties

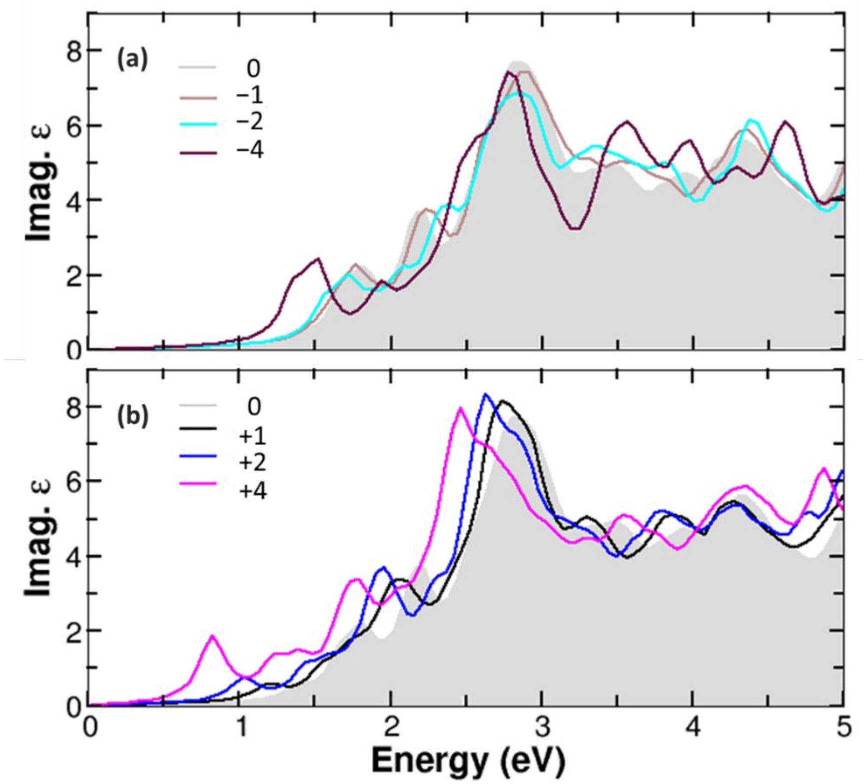

Figure S8. Imaginary part of the macroscopic dielectric function of the $MoS_2/ZrGe_2N_4$ HBL calculated in the independent particle approximation on top of the HSE06 electronic structure under (a) compressive and (b) tensile strain with magnitude reported in the legends. The spectrum of the unstrained system (0) is plotted for reference in both panels as a gray area.
